# Disruption of Nrf2 Enhances Upregulation of Nuclear Factor-*κ*B Activity, Proinflammatory Cytokines, and Intercellular Adhesion Molecule-1 in the Brain after Traumatic Brain Injury

**DOI:** 10.1155/2008/725174

**Published:** 2009-01-25

**Authors:** Wei Jin, Handong Wang, Wei Yan, Lizhi Xu, Xiaoliang Wang, Xiaoning Zhao, Xiaohe Yang, Gang Chen, Yan Ji

**Affiliations:** ^1^Department of Neurosurgery, Jinling Hospital, School of Medicine, Nanjing University, Nanjing 210002, Jiangsu Province, China; ^2^Department of Neurosurgery, Second Affiliated Hospital, School of Medicine, Zhejiang University, Hangzhou 310009, Zhejiang Province, China; ^3^Department of Medical Genetics, School of Medicine, Nanjing University, Nanjing 210093, Jiangsu Province, China; ^4^Department of Oral and Maxillofacial Surgery, Affiliated Stomatology Hospital, School of Medicine, Nanjing University, Nanjing 210008, Jiangsu Province, China

## Abstract

Inflammatory response plays an important role in the pathogenesis of secondary brain injury after traumatic brain injury (TBI). Nuclear factor erythroid 2-related factor 2 (Nrf2) is a key transcription factor that plays a crucial role in cytoprotection against inflammation. The present study investigated the role of Nrf2 in the cerebral upregulation of NF-*κ*B activity, proinflammatory cytokine, and ICAM-1 after TBI. Wild-type Nrf2 (+/+) and Nrf2 (−/−)-deficient mice were subjected to a moderately severe weight-drop impact head injury. Electrophoretic mobility shift assays (EMSAs) were performed to analyze the activation of nuclear factor kappa B (NF-*κ*B). Enzyme-linked immunosorbent assays were performed to quantify the production of tumor necrosis factor-*α* (TNF-*α*), interleukin-1*β* (IL-1*β*), and interleukin-6 (IL-6). Immunohistochemistry staining experiments were performed to detect the expression of intercellular adhesion molecule-1 (ICAM-1). Nrf2 (−/−) mice were shown to have more NF-*κ*B activation, inflammatory cytokines TNF-*α*, IL-1*β* and IL-6 production, and ICAM-1 expression in brain after TBI compared with their wild-type Nrf2 (+/+) counterparts. The results suggest that Nrf2 plays an important protective role in limiting the cerebral upregulation of NF-*κ*B activity, proinflammatory cytokine, and ICAM-1 after TBI.

## 1. INTRODUCTION

Cerebral inflammation plays an important role in the pathogenesis of secondary brain injury following traumatic brain injury (TBI) [1, 2]. Proinflammatory nuclear factor kappa B (NF-*κ*B) signaling pathway has been well documented in previous studies of our laboratory [3, 4]. Increased levels of inflammatory agents with the injured brain, including tumor necrosis factor-*α* (TNF-*α*), interleukin-1*β* (IL-1*β*), interleukin-6 (IL-6), and intercellular adhesion molecule 1 (ICAM-1), are believed to contribute to the cerebral damage [5]. Their mediator NF-*κ*B activation enhances the transcription of proinflammatory cytokines [6], and the cytokines are known to in turn activate NF-*κ*B [7]. The positive feedback is believed to serve to amplify inflammatory signals and exacerbate brain injury after TBI.

Recent researches have demonstrated that nuclear factor erythroid 2-related factor 2 (Nrf2), a key transcription factor that regulates the cellular antioxidant response, plays a broader role in modulating acute inflammatory response [8, 9]. Under basal conditions, Nrf2 is sequestered in the cytoplasm by the cytosolic regulatory protein Keap1. In conditions of oxidative or xenobiotic stress, Nrf2 translocates from the cytoplasm to the nucleus, and sequentially binds to a promoter sequence called the antioxidant response element (ARE), resulting in a cytoprotective response which is characterized by upregulation of a group of antioxidant enzymes and decreased sensitivity to oxidative damage [10–12]. These antioxidant enzymes have also been shown to protect cells against acute inflammatory response [13].

Numerous studies have reported that Nrf2 plays a critical role in counteracting inflammation in a variety of experimental models. Nrf2 protects against allergen-mediated airway inflammation [14], cigarette smoke-induced emphysema [15], dextran sulfate sodium (DSS)-mediated colitis [16], inflammation-mediated colonic tumorigenesis [17], and inflammatory responses during skin wound healing [18]. Furthermore, Nrf2 has also been reported as a crucial regulator of the innate immune response and survival during experimental sepsis [19]. In one of our previous studies, we have demonstrated that TBI could induce Nrf2-ARE pathway activation in brain [20].

Therefore, it may be reasonable to postulate that Nrf2 plays an important role in limiting the cerebral inflammatory response after TBI. In our study, we evaluated the influence of Nrf2 genotype on the cerebral upregulation of NF-*κ*B activity, proinflammatory cytokine, and ICAM-1 after TBI.

## 2. MATERIALS AND METHODS

### 2.1. Animals

Our experiments were conformed to Guide for the Care and Use of Laboratory Animals from National Institutes of Health and approved by the Animal Care and Use Committee of Nanjing University. Breeding pairs of Nrf2-deficient ICR mice were kindly provided by Dr. Thomas W. Kensler (Johns Hopkins University, Baltimore, Md, USA). Homozygous wild-type Nrf2 (+/+) and Nrf2 (−/−)-deficient mice were generated from inbred heterozygous Nrf2 (+/−) mice [10]. Genotypes of Nrf2 (+/+) and Nrf2 (−/−) mice were confirmed by PCR amplification of genomic DNA isolated from the blood. PCR amplification was carried out by using three different primers, 5′-TGGACGGGACTATTGAAGGCTG-3′ (sense for both genotypes), 5′-CGCCTTTTCAGTAGATGGAGG-3′ (antisense for wild-type), and 5′-GCGGATTGACCGTAATGGGATAGG-3′ (antisense for LacZ). Age- and weight-matched adult male mice (6–8 weeks, 28–32 g) were separated into four groups (*n* = 10 per group): group I, sham wild-type (Nrf2 +/+); group II, injured wild-type (Nrf2 +/+); group III, sham-deficient (Nrf2 −/−); group IV, injured-deficient (Nrf2 −/−). The mice of sham and injured groups were subjected to identical anesthetic alone or experimental TBI, respectively. Animals were decapitated at 24 hours following sham or injury. Five mice in each group were sacrificed for electrophoretic mobility shift assay (EMSA) and enzyme-linked immunosorbent assay (ELISA) analysis and the others were for immunohistochemistry study.

### 2.2. Induction of experimental TBI

The mouse model of TBI was employed as described [21] with recent minor modification [22]. The mice were anesthetized by intraperitoneal injection with sodium pentobarbital (50 mg/kg). A round, flat, and 6 mm diameter Teflon impounder was centered between the ears and eyes. TBI was induced by a 100 g weight dropped from a 12 cm height along a stainless steel string, which translated into 1200 g/cm. Brain injury-induced apnea was then treated for 3 minutes with 100% oxygen administration and chest compression to stimulate the respiration. This model is generally associated with 20% of mortality within the first 5 minutes postinjury and no delayed mortality was observed thereafter. After operation procedures, the mice were returned to their cages. Heart rate, arterial blood pressure, and rectal temperature were monitored, and the rectal temperature was kept at 37 ± 0.5°C (physical cooling if required) throughout experiments.

**Figure 1:**
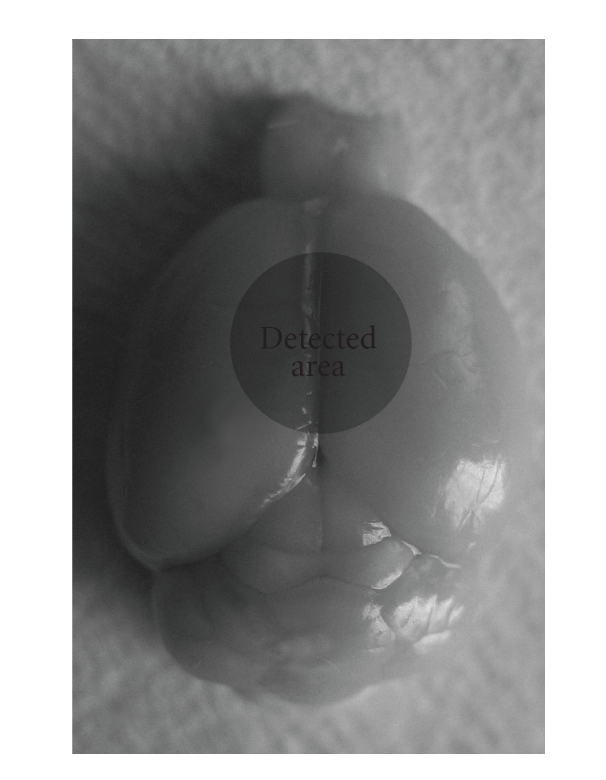
Schematic representation of the area taken for assay.

At the 24 hours following sham or injury, mice were sacrificed for sample collection. For EMSA and ELISA analyses, mice were exsanguinated by cardiac puncture. Cortex tissue was rapidly taken from the fresh brain at the site of lesion (Figure 1), and stored in liquid nitrogen immediately. For immunohistochemistry, mice were perfused with cold saline (4°C), followed by 4% neutral-buffered formalin. The cortex tissue was taken, stored overnight in 4% neutral-buffered formalin, and then embedded in paraffin.

### 2.3. Nuclear protein extract and EMSA

Nuclear protein was extracted and quantified as described [23]. Briefly, frozen brain samples were homogenized in 0.8 mL ice-cold buffer A composed of 10 mmol/L HEPES pH 7.9, 10 mmol/L KCl, 2 mmol/L MgCl_2_, 0.1 mmol/L EDTA, 1 mmol/L dithiothreitol (DTT), and 0.5 mmol/L phenylmethylsulfonyl fluoride (PMSF) (all from Sigma Chemical Co., St. Louis, Mo, USA). The homogenates were incubated on ice for 30 minutes and vortexed for 30 seconds after addition of 50 *μ*L 10% NP-40 (Sigma Chemical Co., Mo, USA). The mixture was then centrifuged for 10 minutes (5000 × g, 4°C). The pellet was suspended in 100 *μ*L ice-cold buffer B composed of 50 mmol/L HEPES pH 7.9, 50 mmol/L KCl, 300 mM NaCl, 0.1 mmol/L EDTA, 1 mmol/LDTT, and 0.5 mmol/L PMSF, and 10% (v/v) glycerol and incubated on ice 30 minutes with frequent mixing. After centrifugation (12000 × g, 4°C) for 15 minutes, the supernatants were collected as nuclear extracts and stored at −70°C for further use. Protein concentration was determined using a bicinchoninic acid assay kit with bovine serum albumin as the standard (Pierce Biochemicals, Rockford, Ill, USA).

EMSA was performed using a commercial kit (Gel Shift Assay System; Promega, Madison, Wis, USA) following the methods in our laboratory [23]. Consensus oligonucleotide probe (5′-AGTTGA GGGGACTTTCCCAGGC-3′) was end-labeled with [*γ*-^32^P]ATP (Free Biotech., Beijing, China) with T4-polynucleotide kinase. Nuclear protein (10 *μ*g) was preincubated in a total volume of 9 *μ*L in a binding buffer, consisting of 10 mmol/L Tris-HCl (pH 7.5), 4% glycerol, 1 mmol/L MgCl_2_, 0.5 mmol/L M EDTA, 0.5 mmol/L DTT, 0.5 mmol/L NaCl, and 0.05 g/L poly-(deoxyinosinic-deoxycytidylic acid) for 15 minutes at room temperature. After addition of the 1 *μ*L ^32^P-labled oligonucleotide probe, the incubation was continued for 20 minutes at room temperature. Reaction was stopped by adding 1 *μ*L of gel loading buffer and the mixture was subjected to nondenaturing 4% polyacrylamide gel electrophoresis in 0.5 × TBE buffer (Tris-borate-EDTA). After electrophoresis was conducted at 390 V for 1 hour, the gel was vacuum-dried and exposed to X-ray film (Fuji Hyperfilm, Tokyo, Japan) at −70°C with an intensifying screed. Levels of NF-*κ*B DNA binding activity were quantified by computer-assisted densitometric analysis.

### 2.4. ELISA analysis

Frozen brain samples were homogenized in 1 mL of buffer containing 1 mmol/L of PMSF, 1 mg/L of pepstatin A, 1 mg/L of aprotinin, and 1 mg/L of leupeptin in PBS solution (pH 7.2) with a glass homogenizer and then centrifuged at 12000 g for 20 minutes at 4°C. The supernatant was then collected and total protein was determined by the Bradford method. The levels of inflammatory cytokines were quantified using enzyme-linked immunosorbent assay (ELISA) kits specific for mouse according to the manufacturers' instructions (TNF-*α* from Diaclone Research, France; IL-1*β*, IL-6 from Biosource Europe SA, Belgium) and previous study of our laboratory [24]. The cytokine contents in the brain samples were expressed as pg per milligram protein.

### 2.5. Immunohistochemical staining

The paraffin-embedded sections (4 *μ*m) were used for Immunohistochemical assay, which was performed with a goat antimouse ICAM-1(CD54) antibody (diluted 1:200, R&D Systems, Inc., Minn, USA), according to previous studies of our laboratory [7]. The sections were incubated with the diluted antibody overnight at 4°C in a humid chamber, washed, and blocked with 1.6% H_2_O_2_ in phosphate-buffered saline (PBS) for 10 minutes. After washing with PBS again, sections were then incubated with biotinylated second antibodies for 1 hour at room temperature. Diaminobenzidine (DAB) was used as chromogen and counterstaining was done with hematoxylin. The number of positive microvessels in each section was counted in 10 microscopic fields (at 100 × magnifications) and averaged for the positively immunostained vessel number of per visual field.

### 2.6. Statistical analysis

Software SPSS 13.0 was used for the statistical analysis. All data were expressed as mean ± SEM, Student's *t*-test was used to analyze the differences between the sham and TBI groups within a single genotype as well as between genotypes. Statistical significance was accepted at *P* < .05.

## 3. RESULTS

### 3.1. EMSA for NF-*κ*B

**Figure 2:**
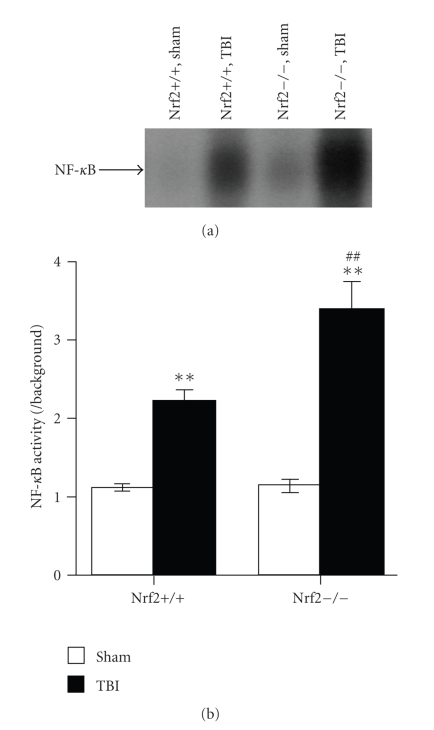
NF-*κ*B activity in the cortex of sham and injured Nrf2 (+/+) and Nrf2 (−/−) mice. (a) Nuclear proteins of brain samples of Nrf2 (+/+) and Nrf2 (−/−) mice were assayed for NF-*κ*B DNA binding activity by EMSA 24 hours after TBI. (b) Quantification of NF-*κ*B DNA binding activity was performed by densitometric analysis. The figure indicates that cerebral NF-*κ*B activity was significantly increased after TBI and was greater in Nrf2 (−/−) mice than in Nrf2 (+/+) mice. Data represents mean ± SEM (*n* = 5 per group). **P < .01 versus sham control of the same genotype. ^*##*^ *P* < .01 versus injured wild-type mice.

NF-*κ*B activation in the nuclear extracts was assessed by EMSA. As shown in Figure 2, low NF-*κ*B banding activity (weak EMSA autoradiography) was observed in sham-operated mice of both genotypes. TBI induced activation of NF-*κ*B in the cortex of both Nrf2 (+/+) and Nrf2 (−/−) mice. Nrf2 (−/−) mice showed an increased susceptibility to TBI-induced activation of NF-*κ*B than their wild-type Nrf2 (+/+) counterparts.

### 3.2. ELISA for inflammatory cytokines

**Figure 3:**
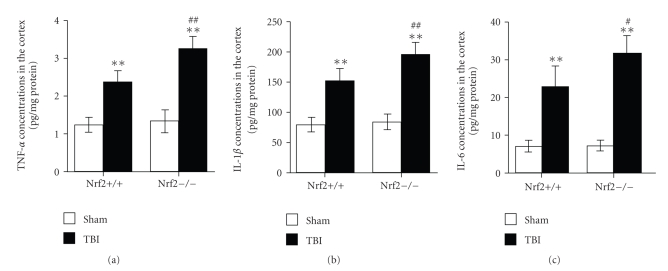
Concentrations of inflammatory cytokines in the cortex of sham and injured Nrf2 (+/+) and Nrf2 (−/−) mice. Concentrations of (a) TNF-*α*, (b) IL-1*β*, and (c) IL-6 were determined by ELISA in the brain samples of Nrf2 (+/+) and Nrf2 (−/−) mice 24 hours after TBI. The figure indicates that concentrations of TNF-*α*, IL-1*β*, and IL-6 in brain were significantly increased after TBI and were greater in Nrf2 (−/−) mice than in Nrf2 (+/+) mice. Data represents mean ± SEM (*n* = 5 per group). **P < .01 versus sham control of the same genotype. ^*#*^ *P* < .05 and ^*##*^ *P* < .01 versus injured wild-type mice.

Concentrations of TNF-*α*, IL-1*β*, and IL-6 in the brain samples were measured by ELISA. As shown in Figure 3, low concentrations of TNF-*α*, IL-1*β*, and IL-6 were observed in sham-operated mice of both genotypes. TBI induced upregulation of TNF-*α*, IL-1*β*, and IL-6 in the cortex of both Nrf2 (+/+) and Nrf2 (−/−) mice. Nrf2 (−/−) mice showed larger increase in cortical levels of TNF-*α*, IL-1*β*, and IL-6 than their wild-type Nrf2 (+/+) littermates after TBI.

### 3.3. Immunohistochemistry for ICAM-1

**Figure 4:**
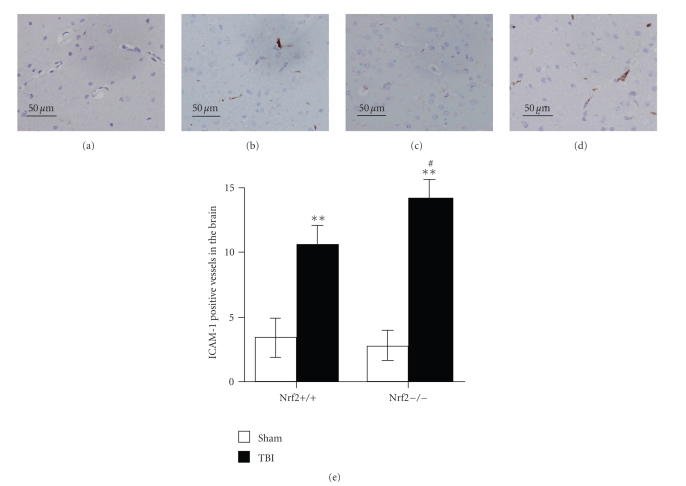
Expression of ICAM-1 in the cortex of sham and injured Nrf2 (+/+) and Nrf2 (−/−) mice. Immunohistochemical staining for ICAM-1 was performed in the cortex tissue sections of Nrf2 (+/+) and Nrf2 (−/−) mice 24 hours after TBI. (a), (c) Sham-operated Nrf2 (+/+) and Nrf2 (−/−) mice showing few ICAM-1-immunostained cerebral microvessels. (b) Injured Nrf2 (+/+) mice showing increased number of ICAM-1 positive vessels. (d) Injured Nrf2 (−/−) mice showing larger increment in the number of ICAM-1 positive vessels compared with injured Nrf2 (+/+) mice. (e) Quantitative analysis showed that the number of ICAM-1 positive vessels in brain was significantly increased after TBI and was greater in Nrf2 (−/−) mice than in Nrf2 (+/+) mice. Data represents mean ± SEM (*n* = 5 per group). **P < .01 versus sham control of the same genotype. ^*#*^ *P* < .05 versus injured wild-type mice.

For assessment of the expression of ICAM-A in the brain after TBI, immunohistochemical study for ICAM-1 was performed. As shown in Figure 4, few ICAM-1-immunostained cerebral microvessels were observed in sham-operated mice of both genotypes. At the 24 hours after TBI, the number of ICAM-1 positive vessels was significantly increased in the cortex of both Nrf2 (+/+) and Nrf2 (−/−) mice. Nrf2 (−/−) mice showed larger increase in the number of ICAM-1 positive vessels than their wild-type Nrf2 (+/+) littermates after TBI.

## 4. DISCUSSION

The most important finding of this study is that Nrf2 (−/−) mice had more inflammatory cytokines TNF-*α*, IL-1*β* and IL-6 production, ICAM-1 expression, and their mediator NF-*κ*B activation in brain after TBI compared with their wild-type Nrf2 (+/+) counterparts. These findings reported here suggest for the first time that Nrf2 may play an important role in limiting the cerebral inflammatory response after TBI through modulating the proinflammatory nuclear factor kappa B (NF-*κ*B) signaling pathway.

Activation of NF-*κ*B signaling pathway has been shown to be central to the pathophysiology of cerebral inflammatory response induced by TBI [6, 7]. NF-*κ*B can be activated by lesion-induced oxidative stress, bacterial endotoxin, and cytokines [25]. The functional importance of NF-*κ*B in inflammation is based on its ability to regulate the promoters of multiple inflammatory genes, including TNF-*α*, IL-1*β*, IL-6, and ICAM-1 [4]. TNF-*α* is reported to be a major initiator of inflammation and is released early after an inflammatory stimulus [26]. IL-1*β* is regarded as the prototypic “multifunctional” cytokine and is induced in a multitude of consequences of cell types [27]. IL-6 is increased after TNF-*α* and is considered to be an important proinflammatory cytokine in contribution to both morbidity and mortality in condition of “uncontrolled” inflammation [28]. ICAM-1, a member of the immunoglobulin superfamily which can be profound induced after cytokine challenge, is important in the recruitment of leukocytes during the inflammatory process [29]. This inflammatory agent network is believed to be important in the generation of acute inflammatory response. NF-*κ*B activation enhances the transcription of proinflammatory cytokines, and the cytokines are known to in turn activate NF-*κ*B [5]. The positive feedback is believed to serve to amplify inflammatory signals and exacerbate brain injury after TBI. In the present study, we evaluated the influence of Nrf2 genotype in the TBI-induced activation of proinflammatory NF-*κ*B signaling pathway in the brain. The results showed that disruption of Nrf2 in mice caused a greater activation of NF-*κ*B signaling pathway which played a critical role in the pathophysiology of cerebral inflammatory response induced by TBI. The observed interplay between Nrf2 and NF-*κ*B signaling corresponds well to the results of study on experimental sepsis, which have demonstrated that Nrf2-deficient mice displayed increased NF-*κ*B activation in response to lipopolysaccharide (LPS) [19].

Although numerous in vivo studies have reported that Nrf2 plays a critical role in counteracting the inflammation in a variety of experimental models [14–19], the findings which we have confirmed and extended in the model of TBI in the present study, the precise mechanism underlying this network is still unclear. Several lines of evidence suggest that Nrf2 regulates the inflammatory response by inhibiting proinflammatory NF-*κ*B activation through maintenance of redox homeostasis. Oxidative stress from reactive oxygen species (ROS) is believed to be involved in the progression of secondary brain injury following TBI [30]. Activation of the NF-*κ*B signaling pathway has been shown to be responsive to excess ROS and is important in the generation of inflammation [19]. Nrf2, as a key antioxidant transcription factor involved in the intracellular antioxidant defense systems, has been shown to play an important role in limiting ROS levels and thereby affect redox-sensitive NF-*κ*B signaling pathway involved in the inflammation [8, 10–12]. The protective function of Nrf2 is mainly mediated by a group of Nrf2-regulated antioxidant and detoxifying enzymes. Therefore, the augmentation of cellular antioxidative or detoxification systems via activation of Nrf2-regulated enzymes resulting in decreased proinflammatory cytokines production and adhesion molecules expression via inactivation of NF-*κ*B represents a possible anti-inflammatory mechanism for the attenuated inflammatory response seen in brains from Nrf2 (+/+) mice but not Nrf2 (−/−) mice after TBI. We then in this study postulated that Nrf2 regulates the TBI-induced cerebral inflammatory response may at least in part through modulating the cerebral redox status and proinflammatory NF-*κ*B signaling pathway. Additional work is necessary to elucidate the whole mechanisms involved in these complicated networks.

In summary, this present study showed that Nrf2 plays a protective role in TBI-induced cerebral upregulation of inflammatory agents in mice. We found that Nrf2 (−/−) mice are more susceptible to TBI-induced cerebral NF-*κ*B activation, inflammatory cytokine TNF-*α*, IL-1*β* and IL-6 production, and ICAM-1 expression, which then contributed to exacerbated brain injury after TBI. To the best of our knowledge, this is the first study that elucidates the interplay between Nrf2 and proinflammatory NF-*κ*B signaling pathway in the brain following TBI. These findings raise the possibility that Nrf2 will be a new therapeutic target for the treatment TBI.
